# Bacterial diversity along a 2600 km river continuum

**DOI:** 10.1111/1462-2920.12886

**Published:** 2015-06-11

**Authors:** Domenico Savio, Lucas Sinclair, Umer Z. Ijaz, Juraj Parajka, Georg H. Reischer, Philipp Stadler, Alfred P. Blaschke, Günter Blöschl, Robert L. Mach, Alexander K. T. Kirschner, Andreas H. Farnleitner, Alexander Eiler

**Affiliations:** ^1^ Centre for Water Resource Systems (CWRS)Vienna University of TechnologyViennaAustria; ^2^ Research Group Environmental Microbiology and Molecular EcologyInstitute of Chemical EngineeringVienna University of TechnologyViennaAustria; ^3^ Institute of Hydraulic Engineering and Water Resource ManagementVienna University of TechnologyViennaAustria; ^4^ Institute for Water Quality, Resource and Waste ManagementVienna University of TechnologyViennaAustria; ^5^ Department of Ecology and Genetics, LimnologyScience for Life LaboratoryUppsala UniversityUppsalaSweden; ^6^ School of EngineeringUniversity of GlasgowGlasgowUK; ^7^ Interuniversity Cooperation Centre Water and Health, www.waterandhealth.atMedical University of ViennaViennaAustria; ^8^ Institute for Hygiene and Applied Immunology, Water HygieneMedical University of ViennaViennaAustria

## Abstract

The bacterioplankton diversity in large rivers has thus far been under‐sampled despite the importance of streams and rivers as components of continental landscapes. Here, we present a comprehensive dataset detailing the bacterioplankton diversity along the midstream of the Danube River and its tributaries. Using 16S rRNA‐gene amplicon sequencing, our analysis revealed that bacterial richness and evenness gradually declined downriver in both the free‐living and particle‐associated bacterial communities. These shifts were also supported by beta diversity analysis, where the effects of tributaries were negligible in regards to the overall variation. In addition, the river was largely dominated by bacteria that are commonly observed in freshwaters. Dominated by the acI lineage, the freshwater SAR11 (LD12) and the *P* *olynucleobacter* group, typical freshwater taxa increased in proportion downriver and were accompanied by a decrease in soil and groundwater‐affiliated bacteria. Based on views of the meta‐community and River Continuum Concept, we interpret the observed taxonomic patterns and accompanying changes in alpha and beta diversity with the intention of laying the foundation for a unified concept for river bacterioplankton diversity.

## Introduction

Streams and rivers link terrestrial and lentic systems with their marine counterparts and provide numerous essential ecosystem services. They supply drinking water, are used for irrigation, industry and hydropower and serve as transport routes or for recreation. Of general importance is the role of lotic systems in biogeochemical nutrient cycling. Until recently, rivers and streams were mainly considered as pipes shuttling organic material and nutrients from the land to the ocean (Cole *et al*., 2007). This view has begun to change as lotic and lentic systems are now considered more akin to ‘leaky funnels’ in regard to the cycling of elements. Indeed, they play an important role in the temporary storage and transformation of terrestrial organic matter (Ensign and Doyle, 2006; Cole *et al*., 2007; Withers and Jarvie, 2008; Battin *et al*., 2009). As a result of recognizing the specific role of streams and rivers in the carbon cycle (Richey *et al*., 2002; Battin *et al*., 2009; Raymond *et al*., 2013), the study of the diverse processes ongoing in the water column and sediments of lotic networks has received increasing interest (Kronvang *et al*., 1999; Beaulieu *et al*., 2010; Seitzinger *et al*., 2010; Aufdenkampe *et al*., 2011; Benstead and Leigh, 2012; Raymond *et al*., 2013).

When attempting to model the mechanisms of nutrient processing in freshwater systems, bacteria are regarded as the main transformers of elemental nutrients and viewed as substantial contributors to the energy flow (Cotner and Biddanda, 2002; Battin *et al*., 2009; Findlay, 2010; Madsen, 2011). However, in the case of open lotic systems such as rivers, there remains a lack of knowledge concerning the diversity of bacterial communities (Battin *et al*., 2009). For example, up until the present day there is no agreement on the distinctness of river bacterioplankton communities from that of other freshwater systems or the variability of its diversity along entire river networks.

Until recently, it was only known that the most abundant taxa comprising riverine bacterioplankton seem to resemble lake bacteria and can thus be regarded as ‘typical’ freshwater bacteria (Zwart *et al*., 2002; Lozupone and Knight, 2007; Newton *et al*., 2011). In particular, bacteria affiliated with the phyla *Proteobacteria* (particularly *Betaproteobacteria*), *Actinobacteria*, *Bacteroidetes, Cyanobacteria* and *Verrucomicrobia* were found to dominate the bacterial communities in rivers (Crump *et al*., 1999; Zwart *et al*., 2002; Cottrell *et al*., 2005; Winter *et al*., 2007; Lemke *et al*., 2008; Mueller‐Spitz *et al*., 2009; Newton *et al*., 2011; Liu *et al*., 2012). A recent metagenome study corroborates a general dominance of the phyla *Proteobacteria* and *Actinobacteria*, and more specifically the clear dominance of the cosmopolitan freshwater lineage acI of the phylum *Actinobacteria* in the Amazon river (Ghai *et al*., 2011). The dominance of *Actinobacteria* and *Proteobacteria* in riverine bacterioplankton was also confirmed in three recent high‐throughput sequencing studies on the Upper Mississippi River (USA; Staley *et al*., 2013), the Yenisei River (RUS; Kolmakova *et al*., 2014) and the River Thames (UK; Read *et al*., 2015). Staley and colleagues (2013) were the first to suggest a persistent and ubiquitous ‘core bacterial community’ along a river stretch. Read and colleagues (2015) examined the longitudinal development of the bacterioplankton community at 23 sites along the river network of the 9948 km^2^ Thames basin. They found a shift from a *Bacteroidetes*‐dominated community in the headwaters to an *Actinobacteria*‐dominated community in the lower reaches near the river mouth and location of the sampling point in the river network to be the most predictive parameter. These patterns they interpreted as evidence for ecological succession along the river continuum.

**Figure 1:**
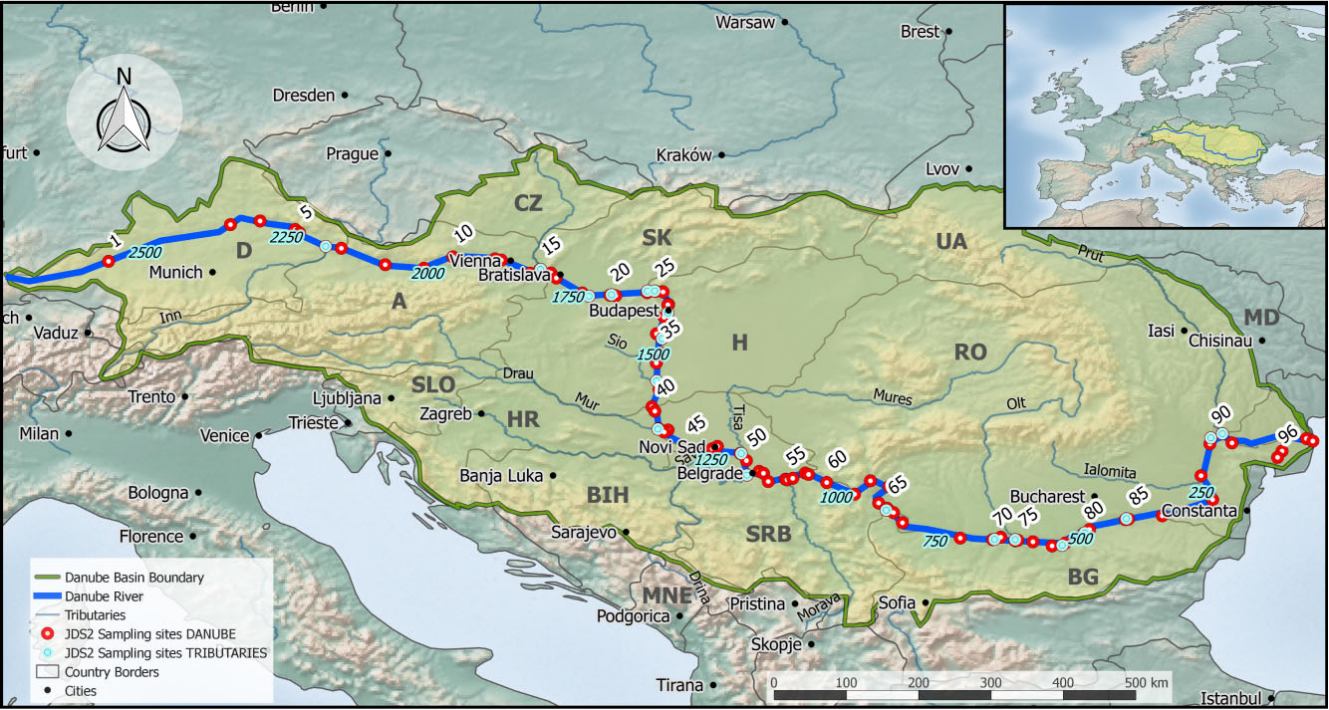
Overview and detailed map of the Danube river catchment showing all sampling sites during the Joint Danube Survey 2; red dots indicate sampling points in the midstream of the Danube River; blue dots represent sampling points in tributaries before merging with the Danube River. Blue‐shaded font indicates official numbering of river kilometres, starting with rkm 2600 at the uppermost site to rkm 0 at the river mouth. Country abbreviations and large cities are written in black. The map was created using Quantum GIS (Quantum GIS Development Team, 2011).

However, the existing studies focused on relatively small river basins and/or a small number of sampling sites. In large river basins, however, one would expect that the spatial patterns of bacterial community compositions manifest themselves more clearly than in small ones due to the larger contrast in environmental conditions. In this paper, we analyse the results from a second‐generation sequencing experiment by separately investigating the free‐living and particle‐associated bacterioplankton communities along 96 sites in the network of the entire Danube basin (Fig. 1). The Danube River is 2780 km in length and drains a catchment area of 801 000 km^2^ with 83 million inhabitants (Schmedtje *et al*., 2004; Sommerwerk *et al*., 2010). Based on our results, we propose that the bacterioplankton communities in the midstream of such a large river develop gradually and increasingly independent from tributary and riparian influence as a result of the interplay between dispersal‐facilitated (‘mass effects’) and environmental condition‐based sorting (‘species sorting’; Leibold *et al*., 2004; Crump *et al*., 2007; 2012). Moreover, we argue that these processes, represented in the meta‐community concept, can be linked to the River Continuum Concept (RCC; Vannote *et al*., 1980), which explains the role of the hydrological flow conditions, the riparian zone, substrate and food as important factors in determining community structures along entire river systems.

## Results

### Selected environmental and geomorphological parameters

In total, more than 280 individual parameters, including chemical, microbiological, ecotoxicological, radiological and biological parameters were investigated within the Joint Danube Survey 2 (Liska *et al*., 2008). Alkalinity, pH, concentration of nitrate as well as dissolved silicates exhibited a gradually decreasing trend along the river as previously described by Liska and colleagues (2008) and illustrated in Fig. S1D–G. Total phytoplankton biomass (Chla) showed a peak between river kilometre 1481 and 1107 (sites 38–55) with total bacterial production following a similar trend, whereas total suspended solid concentration increased considerably in the last 900 kilometres before reaching the Black Sea (Fig. S1H–J).

Several geomorphological measures were calculated such as ‘river kilometre’, ‘catchment area’, ‘mean dendritic stream length’ and ‘cumulative dendritic distance upstream’ and were compared with each other. For example, ‘mean dendritic stream length’ correlated very closely with ‘river kilometre’ (as defined by the distance to mouth; linear model R^2^ = 0.98; *P* < 0.001; Fig. S1A). In contrast, ‘cumulative dendritic distance upstream’ correlated almost perfectly with ‘catchment area’ (linear model R^2^ > 0.99; *P* < 0.001; Fig. S1C). From a hydrological point of view, it can be argued that ‘mean dendritic stream length’ is a better proxy of stream residence time than ‘cumulative dendritic distance upstream’, as it represents the average travel time of a drop of water, assuming randomly distributed spring discharges and constant flow velocities in the river system (Rodriguez‐Iturbe *et al*., 2009).

### Core microbial community

**Figure 2:**
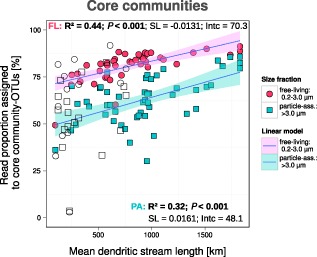
Gradual development of the read proportions assigned to the operationally defined ‘core communities’ of the free‐living and particle‐associated fraction (all OTUs occurring in at least 90% of all Danube River samples of the respective size fraction) along ‘mean dendritic stream length’. Round symbols represent samples from the free‐living size fraction (0.2–3.0 μm); squares represent samples from the particle‐associated size fraction (> 3.0 μm). Red dots indicate samples from the free‐living size fraction (0.2–3.0 μm) of the Danube River only; blue squares indicate samples from the particle‐associated fraction (> 3.0 μm) of the Danube River; open dots and squares represent tributary samples from the free‐living and particle‐associated size fraction, respectively; dark blue lines indicate fitted linear models with confidence intervals of 0.95 in red and blue for the respective size fraction of the Danube River samples. Detailed regression statistics for the core community development in the Danube River (exclusive tributary samples) are shown in the figure.

In total, sequencing resulted in 1 572 361 sequence reads (further referred to as ‘reads’) after quality filtering and clustered into clustering into 8697 bacterial operational taxonomic units (OTUs). The majority of bacteria‐assigned OTUs (4402 out of 8697) were only represented by less than 10 reads in the entire dataset. As a consequence, 3243 of 8697 OTUs (∼37%) were present in only one to four samples, and an additional 2219 OTUs (∼26%) were present in as few as five to nine samples. Besides these rare OTUs, the core community of the Danube River, as operationally defined by all OTUs that appeared in at least 90% of all Danube River samples, comprised 89 OTUs in the free‐living bacterioplankton (0.2–3.0 μm) and 141 OTUs in the particle‐associated fraction (> 3.0 μm). On average, 81% of all reads of the free‐living river community and 63% of all reads of the particle‐associated river community were part of their respective core community. The relative abundance of the core communities in both fractions increased significantly towards the river mouth with similar slopes revealed by regression analysis (Fig. 2).

### Variability in river bacterioplankton alpha diversity

**Figure 3:**
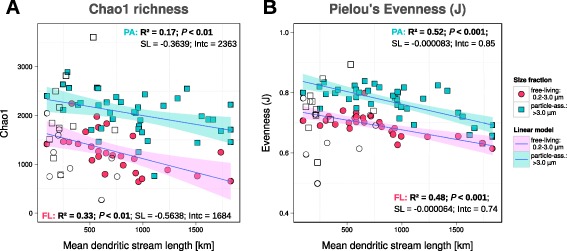
The gradual development of (A) the bacterial richness (Chao1) and (B) Pielou's evenness (J) along the ‘mean dendritic stream length’ at each sampling site; red dots indicate samples from the free‐living size fraction (0.2–3.0 μm) of the Danube River only (*n* = 27); blue squares indicate samples from the particle‐associated fraction (> 3.0 μm) of the Danube River (*n* = 40); open dots and squares represent tributary samples from the free‐living and particle‐associated size fraction, respectively; dark blue lines indicate fitted linear models with confidence intervals of 0.95 in red and blue for the respective fraction of Danube River samples. Detailed regression statistics for Danube River samples (exclusive tributary samples) are shown in the figure.

To investigate alpha diversity, we calculated the Chao1 richness estimator and Pielou's evenness index for both size fractions after rarefying all samples down to 7000 reads and discarding 36 samples with fewer reads. We observed the highest diversity of all samples in the upstream part of the Danube River, representing medium‐sized stream reaches according to the RCC definition. Richness and evenness then gradually decreased downstream in both size fractions (Fig. 3A and B) as confirmed by regression analysis using ‘mean dendritic stream length’ as well as ‘river kilometre’, ‘cumulative dendritic distance upstream’ and ‘catchment area’ (Table S1). For bacterial richness, the regressions revealed very similar slopes for the free‐living and the particle‐associated fractions (Fig. 3A). Besides the observed trends in the midstream communities of the Danube River, the tributary communities frequently formed outliers with considerably lower richness and evenness (Fig. 3A and B).

Among the two size fractions, the estimated richness was consistently higher in the particle‐associated communities than in the free‐living fraction (Wilcoxon rank sum test; *P*‐value < 0.001) with means of 2025 OTUs and 1248 OTUs, respectively (Fig. 3A). Similar observations were reported in studies on (coastal) marine environments as well as lentic freshwater environments (Bižić‐Ionescu *et al*., 2014; Mohit *et al*., 2014), ascribing the higher alpha diversity in the particle‐associated communities to the high heterogeneity in the particle microenvironment. A large spectrum of niches can be provided by the high heterogeneity among particles in rivers including mobilized sediments, living organisms such as planktonic algae or zooplankton and detritus derived from terrestrial and aquatic sources. Bižić‐Ionescu and colleagues (2014) even suggested that the presence of diversely colonized particles of different age, origin and composition can be described based on the observation of a higher richness in the particle‐associated fraction.

### Variability in river bacterioplankton beta diversity

**Figure 4:**
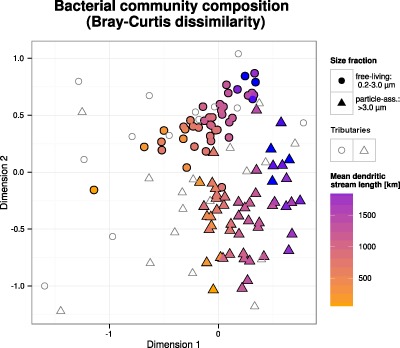
Non‐metric multidimensional scaling plot of the compositional dissimilarities between communities (Bray–Curtis dissimilarities) of all samples of the Danube River and its tributaries. The stress value of NMDS was 0.17. Dots represent free‐living bacterial communities (0.2–3.0 μm); triangles display particle‐associated bacterial communities (> 3.0 μm). Open symbols represent tributary samples, whereas full symbols indicate communities in the Danube River. The gradient from orange to blue via purple indicates the ‘mean dendritic stream length’ at the respective sampling site in the Danube River.

While communities of both size fractions in the Danube River covary significantly with alkalinity, nitrate concentration and dissolved silicates, the particle‐associated communities additionally covaried highly significantly with total bacterial production, nitrite concentrations, phytoplankton biomass and total suspended solids (Table 1). These correlations indicate that chemical properties may determine bacterioplankton community composition. Nevertheless, the observed significant relationship between community composition and ‘mean dendritic stream length’ (Table 1) emphasizes the underlying role of stream travel times. Still, as tributary samples were scattered in the multidimensional space (Fig. 4), the midstream Danube River communities are suggested to develop independently from tributary communities, which is further supported when depicting tributaries according to their position of confluence within the Danube River (see Fig. S2).

**Table 1:** Summary statistics of correspondence between environmental variables and the projections of bacterioplankton community samples in the NMDS ordination based on either free‐living or particle‐associated size fractions for the Danube River samples only (left) and tributary samples included (right). The results were obtained using the function ‘envfit’ included in the R‐package ‘vegan’ (Oksanen *et al*., 2013)

	Danube River only		Danube River and tributary samples	
	Free living	Particle associated	Free living	Particle associated
	R^2^	R^2^	R^2^	R^2^
Official Danube River kilometre^a^ (for tributaries rkm at confluence)	0.840***	0.832***	0.241***	0.236***
Official Danube River kilometre^a^ (calculated for tributaries^b^)	0.840***	0.832***	0.628***	0.574***
**Mean dendritic stream length (water residence time)**	0.799***	0.808***	0.620***	0.579***
Median dendritic length	0.752***	0.773***	0.594***	0.554***
Catchment size	0.766***	0.803***	0.559***	0.524***
Cumulated dendritic distance	0.772***	0.807***	0.568***	0.527***
Nitrate	0.677***	0.538***	0.294**	0.099*
Alkalinity	0.605***	0.430***	0.329**	0.159*
Silicates dissolved	0.500***	0.589***	0.200*	0.167*
Total bacterial production	0.159*	0.440***	0.399**	0.445***
Bacterial production filtered fraction	0.059	0.468***	0.379*	0.425***
Nitrite	0.220*	0.283***	0.039	0.160*
Phytoplankton biomass (Chla)	0.016	0.396***	0.039	0.222**
Total suspended solids	0.143*	0.347***	0.117	0.166**
pH	0.140	0.175**	0.088	0.031
Water temperature	0.100	0.029	0.103	0.002
Organic nitrogen	0.055	0.131*	0.032	0.091*
Conductivity	0.200*	0.107	0.131*	0.418***
Orthophosphate phosphorus	0.083	0.075	0.583***	0.337***
Ammonium	0.073	0.026	0.445**	0.301**
Total phosphorus	0.057	0.063	0.078	0.400***
Dissolved oxygen	0.052	0.007	0.248**	0.069

Significant codes: ***≤ 0.001 **≤ 0.01

*≤ 0.05.

a rkm 2600 = upstream region [near Ulm (DE)]; rkm 0 = river mouth (Black Sea).

b To obtain the corresponding 'Danube river kilometres' according to the official numbering (see a) for tributaries, the length of tributaries was calculated by subtracting the official length of tributaries (Schmedtje *et al*., 2004) from the official length of the Danube River (2780 km; Schmedtje *et al*., 2004).

In addition to the observation that (i) tributary bacterioplankton communities did not follow the general patterns of midstream Danube River communities and often formed outliers in the ordination space, other visual impressions from the non‐metric multidimensional scaling (NMDS) are that (ii) there is a distinction in community composition between the two size fractions as confirmed by permutational multivariate analysis of variance (PERMANOVA) analysis (R^2^ = 0.156, *P*‐value < 0.01), and (iii) there appears to be synchrony in the community changes of the two size fractions along the river's course, which we statistically verified using a Procrustes test (r = 0.96, *P* < 0.001). Furthermore, (iv) a permutation test on the beta dispersion values between the samples of each size fraction revealed a higher variability (by a factor of 0.06) in the > 3.0 μm fraction when compared with the 0.2–3.0 μm fraction (*P*‐value = 0.002) (see Fig. S3). This supports the idea of a larger heterogeneity in niche availability in the particle‐associated communities based on higher diversity in particle age and origin.

### Typical river bacterioplankton

**Figure 5:**
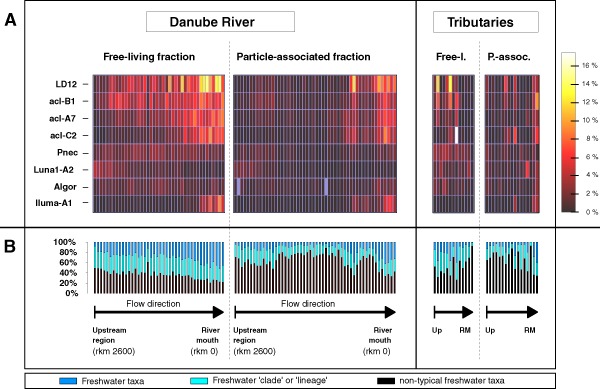
A heat map (A) revealing the dynamics of the eight most abundant typical freshwater tribes along the Danube River defined according to Newton and colleagues (2011). The gradient from black red via yellow to white indicates the relative quantitative contribution of the respective tribe to all sequence reads in any one sample, with a maximum of 16%. Panel (B) displays the overall contribution of typical freshwater tribes (dark blue) as well as clades and lineages (turquoise) according to the definition by Newton and colleagues (2011) to the river bacterioplankton amplicon sequences along the river; black bars represent reads that could not be matched to sequences of the used freshwater database (Newton *et al*., 2011) neither on tribe, clade or lineage‐level (named ‘non‐typical freshwater taxa’). ‘Freshwater taxa’ and ‘freshwater clade or lineage’ represent all reads that could be matched to sequences of the used freshwater database at the respective similarity level. Samples from the Danube River as well as the investigated tributaries are arranged from left to the right, with increasing distance from the source and separated according to the respective size fractions.

Along the river, the bacterioplankton community was dominated by *Actinobacteria*, *Proteobacteria*, *Bacteroidetes*, *Verrucomicrobia* and candidate division OD1, with an increasing proportion of reads assigned to the phylum *Actinobacteria* in the free‐living size fraction downriver (Fig. S4). In contrast, reads assigned to *Bacteroidetes* decreased significantly along the river in the free‐living fraction. In the particle‐associated fraction, these trends in phylum composition were less pronounced. In addition to assigning reads to the phylum level, we taxonomically annotated all 9322 OTUs using similarity searches against the database of freshwater bacteria developed by Newton and colleagues (2011). The analysis revealed that up to 80% of the free‐living and more than 65% of the particle‐associated bacterial population inhabiting the midstream Danube River communities could be assigned to previously described freshwater taxa (Fig. 5B). In particular, these included representatives of the LD12‐tribe belonging to the subphylum of *Alphaproteobacteria*, as well as the acI‐B1‐, acI‐A7‐ and acI‐C2‐tribes belonging to the phylum *Actinobacteria*. Such dominance of few freshwater taxa in riverine bacterioplankton communities (see also Zwart *et al*., 2002; Lozupone and Knight, 2007; Newton *et al*., 2011; Read *et al*., 2015) corroborates the idea that river bacterioplankton resembles those of lakes.

Interestingly, in the free‐living size fraction, we observed a clear increase in the relative abundance of the four above‐mentioned tribes towards the river mouth (Fig. 5A), contributing up to 35% of the community. The increasing relative abundance of these four tribes was accompanied by a general increase in relative abundance of OTUs matching other freshwater tribes, lineages or clades according to Newton and colleagues (2011) as depicted in Fig. 5B. In contrast, the number of OTUs not matching any sequence of the freshwater database either at tribe, clade or at lineage‐level decreased (Fig. 5B; labelled ‘non‐typical freshwater taxa’), suggesting that these OTUs may originate from non‐aquatic sources. In the particle‐associated fraction, typical freshwater taxa were less common (Fig. 5B).

**Figure 6:**
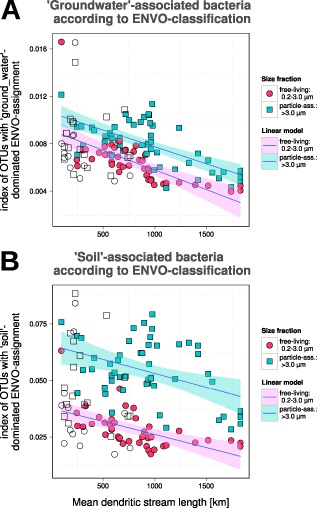
Results from the SEQ env analysis scoring sequences according to their environmental context (EnvO; environmental ontology). The Y‐axis represents the proportion of (A) ‘groundwater’ and (B) ‘soil’ terms associated with sequence reads per sample along the ‘mean dendritic stream length’ at each sampling site (X‐axis). Red dots indicate Danube River samples of the 0.2–3.0 μm size fraction (*n* = 42), and blue squares indicate samples from the > 3.0 μm size fraction (*n* = 52). Open dots and squares represent tributary samples of the free‐living and particle‐associated size fractions respectively. Dark blue lines represent fitted linear models for the Danube River samples with confidence intervals of 0.95 in red and blue for the respective fractions. Detailed regression statistics are given in Table S1.

To confirm the non‐aquatic origin of certain OTUs, we first blasted a representative for each of the 8697 bacterial OTUs against the NCBI‐Nucleotide database; next, any environmental descriptive terms occurring in the search results were retrieved and classified according to the Environmental Ontology (EnvO; Buttigieg *et al*., 2013) terminology. Permutational analysis of variance of the EnvO‐classified data revealed a significant difference in variance between the two size fractions (PERMANOVA; R^2^ = 0.42, *P* < 0.0001), supporting a high variability in origin and particle age. Restricting the analysis to particular EnvO terms such as ‘groundwater’ and ‘soil’ suggests that the proportion of bacteria potentially originating from these sources decreased towards the river's mouth (Fig. 6A and B), while ‘river’, ‘lake’ and ‘epilimnion’ terms exhibited increasing trends downriver (Fig. S5A–C). In particular, we could only identify four OTUs receiving a classification that was dominated by the ‘river’ term.

As with every homology‐based assignment, SEQenv results are affected by database entries; for example the under‐representation of entries from rivers compared with lakes likely biases against the ‘river’ term. Thus, a larger number of typical river bacterioplankton may exist than detected by our analysis.

## Discussion

The tremendous diversity within the microbial communities inhabiting all types of environments is being revealed by a rapidly increasing number of studies applying high‐throughput sequencing technologies (e.g. Sogin *et al*., 2006; Andersson *et al*., 2009; Galand *et al*., 2009; Eiler *et al*., 2012; Peura *et al*., 2012). At the same time, many mechanisms modulating this diversity have been suggested including ‘mass effects’ and ‘species sorting’, which vary widely in importance depending on the environment (Leibold *et al*., 2004; Besemer *et al*., 2012; Hanson *et al*., 2012; Lindström and Langenheder, 2012; Szekely *et al*., 2013).

Regarding bacterioplankton in large river networks, we propose that the highest diversity exists in headwaters, and from thereon decreases towards river mouths. This, we argue, results from the inoculation of bacterioplankton by advection from surrounding environments (i.e. soil and groundwater) in the headwaters as supported by our SEQenv results and previous studies (Crump *et al*., 2007; 2012; Besemer *et al*., 2012; 2013). This initial pervasive impact from the riparian zone on headwaters (‘mass effects’) can simply be justified as follows: (i) in lotic environments, bacterioplankton is transported primarily passively, (ii) the large contact zone of small headwaters (large surface‐area‐to‐volume ratio) with the surrounding environment (soil and groundwater) facilitates the contribution of allochthonous bacteria to the river community (Crump *et al*., 2007; 2012; Besemer *et al*., 2012), (iii) these source environments of inoculation (soils and groundwater) harbour a much higher diversity than aquatic communities (e.g. Crump *et al*., 2012), and (iv) these newly introduced allochthonous bacteria should be at least temporarily capable of proliferating in their new lotic environment, making them constitutive members of the community. Overall, this process of allochthonous input can be described by the so‐called ‘mass‐effect’, where dispersal of organisms exceeds the rate of local extinction (Leibold *et al*., 2004; Crump *et al*., 2007; 2012).

Flowing downriver, with increasing river width and decreasing ‘riparian influence’, we propose that ‘species‐sorting’ progressively prevails over ‘mass effects’ in shaping the bacterioplankton composition. This is supported by the increase of the core communities' relative abundance in both size fractions (Fig. 2) as well as the rapidly decreasing number of first‐time occurrences of OTUs from upstream to downstream (Fig. S6). The associated progressive rise of few and more competitive taxa is further supported by the observed simultaneous decrease in evenness together with bacterial richness in both size fractions downriver (Fig. 3A and B). Finally, the decrease of cell volumes along the Danube River (Velimirov *et al*., 2011) as well as a rise of typical freshwater bacteria (Fig. 5A and B), representing small cells with oligotrophic lifestyles (Salcher *et al*., 2011; Garcia *et al*., 2013), provides further evidence for the increasing importance of ‘species‐sorting’. The increasing resemblance with lake communities (Fig. S5B and C) further corroborates the idea that lake and river bacterioplankton resemble each other.

The interplay between ‘species sorting’ and ‘mass effects’ is fundamental to the meta‐community concept (Leibold *et al*., 2004) and has previously been used to explain the decreasing diversity along a path from upslope soils via headwater streams to a final lake (Crump *et al*., 2012). The latter study suggested advection of soil bacteria to strongly influence the receiving water bodies due to the ‘mass effect’ of dispersing organisms exceeding the rate of ‘species sorting’ (Crump *et al*., 2012). For large river networks, however, this raises the question of when ‘species sorting’ will exceed ‘mass effects’. In contrast to ‘mass effects’, ‘species sorting’ presumes that bacterial growth rates have to be shorter than the residence time in the stream. Assuming a maximum bacterial growth rate of about 1d^−1^ (as calculated for the bulk communities along the Danube River with a maximum and mean of 0.92d^−1^ and 0.18d^−1^, respectively) and considering an estimated in‐stream residence time for the Danube River of 32 days (Velimirov *et al*., 2011), this provides sufficient time for ‘successful’ species sorting.

In contrast to Crump and colleagues (2012), Read and colleagues (2015) put little emphasis on the ‘mass effects’ of the riparian zone and, instead, explained the downstream shift in the bacterial communities along the River Thames as the result of ecological succession. They argued that sampling sites not only represented a spatial distribution but also a time series where downstream sites with longer water residence times contain older river water and a planktonic community that is in the later stages of ecological succession. However, a concept like ecological succession might not be the most suited to describe bacterioplankton diversity patterns in the Danube basin which is much larger, and the residence times are about five times as long when compared with the River Thames. First, we could not observe an initial active growth and propagation of new pioneer species (often r‐strategists) as it would be expected during ecological succession, but solely a decrease in proportions of *Bacteroidetes*‐affiliated cells. Second, the data clearly showed a decrease in alpha diversity, both evenness and richness, downriver. Assuming a dominance of fast‐growing r‐strategists in the highly dynamic communities of the upstream reaches, one would expect low evenness in these reaches. Moreover, highest production rates, as anticipated for a dominance of r‐strategists, were not observed in the upstream reaches, but the middle section of the Danube River [between river kilometre (rkm) 1632‐1071; Velimirov *et al*., 2011 ]. Besides, elevated production rates in the uppermost section were largely assignable to particle‐associated bacteria (Velimirov *et al*., 2011), indicating high‐quality particulate substrate originating from the riparian zone.

Instead of viewing the community assembly in headwaters as the colonization of a lifeless area (primary succession) or the development following a disturbance of an established community (secondary succession), we simply regard streams as part of the hydrological cycle such as soil waters and interstitial groundwaters, in which bacteria are transported passively. Thus, we conclude that dispersal‐based concepts are more appropriate for describing the community assembly at the transition between the different compartments. The Danube data suggest that the ‘mass effects’ of soil and groundwater bacteria across the streambed contact zone are important processes, as reflected in the decreasing proportion of soil and groundwater‐associated bacteria. This decrease of the effect of the streambed contact zone can be illustrated by comparing the uppermost and lowest reaches of the survey, using the ratio of wetted perimeter and cross‐sectional area as a measure of river bed contact zone. At the Upper Danube near Ulm, Germany, an average discharge of 40 m^3^ s^−1^ translates into a ratio of about 0.83 m^−1^ while at the Lower Danube near Reni, Romania, an average discharge of 6300 m^3^ s^−1^ translates into a ratio of 0.056 m^−1^. This means that within the sampled reach, the effect of the contact zone decreases by more than 10‐fold downriver.

Taken together, the findings of this paper and the existing literature (Besemer *et al*., 2012; 2013; Crump *et al*., 2012; Staley *et al*., 2013) suggest that the diversity of bacterioplankton decreases from headwaters to the river mouth due to the decreasing importance of the ‘riparian influence’. This is consistent with the important role the RCC assigns to the riparian zone as well as to the physical drivers such as river flow and wetted perimeter. Moreover, by referring to dissolved organic matter (DOM) in terms of quality, proposing highest DOM diversity in headwaters and a downstream export of more refractory compounds, the RCC also allows us to incorporate a proposed DOM (‘food’)‐based ‘species sorting’, leading to an oligotrophic freshwater bacteria‐dominated community with increasing water residence time. Analogously, an increase in the abundance of a few, more competitive species along the river is implied in the RCC for macroorganisms from medium‐sized reaches towards river mouths (Vannote *et al*., 1980). However, to further develop and incorporate bacterioplankton into the RCC, future studies should address the role of DOM quality changes as a function of catchment characteristics as well as seasonal changes in physical and chemical features of riverine systems.

In summary, the data from the Danube survey along a 2600 km river continuum indicated that bacterial richness and evenness gradually declined downriver in both the free‐living and particle‐associated bacterial communities, resulting in an increase in relative abundance of typical freshwater taxa downstream. The decreasing influence of soil and groundwater bacteria downstream suggests the RCC as a valid interpretive framework which stipulates a continuous gradient of physical conditions that elicit a series of biological responses, resulting in consistent patterns of community structure and function along the river system.

## Experimental procedures

### Supporting data

Within the frame of the second Joint Danube Survey (JDS2), a wide range of chemical and biological parameters was collected (Liska *et al*., 2008). All data, sampling methods as well as analytical methods, are made publicly available via the official website of the International Commission for the Protection of the Danube River (http://www.icpdr.org/wq‐db/) and data used in this study is provided in Table S3.. Selected data from Joint Danube Survey (JDS) 1 and 2 were published previously in several studies (Kirschner *et al*., 2009; Janauer *et al*., 2010; Velimirov *et al*., 2011; Von der Ohe *et al*., 2011). Geomorphological parameters including ‘catchment area’, ‘mean dendritic stream length’ and, for comparison, ‘cumulative dendritic distance upstream’ were calculated based on data from the Catchment Characterisation and Modelling (CCM) River and Catchment Database, version 2.1 (De Jager and Vogt, 2010). The ‘mean dendritic stream length’ was calculated by first identifying the stream paths from all springs in the catchment area upstream a sampling site to that sampling site. The lengths of these paths were than averaged. This implies that the shared reaches were counted multiple times consistent with the movement of water drops in the river basin. This parameter gives the average flow distance (assuming the spring discharges are randomly distributed in the catchment) and therefore the average residence time in the stream (assuming constant flow velocities). In contrast, the ‘cumulative dendritic distance upstream’ was calculated as the sum of the blue lines on the map (counting the lengths below confluences only once) which gives a geometric parameter indicative of the drainage density, but not of residence times.

Additionally, as a measure of the river bed contact zone, the ratio of wetted perimeter and cross‐sectional area was calculated for the long‐term average discharge. Both the wetted perimeter and the areas are obtained from bathymetric surveys. The product of the area and the flow velocity gives the discharge. Here, typical values of the ratio were chosen for a short reach upstream the sampling sites.

### Study sites and sample collection

Samples were collected within the frame of the JDS 2 project in 2007. The overall purpose of the Joint Danube Surveys is to produce a comprehensive evaluation of the chemical and ecological status of the entire Danube River on the basis of the European Union Water Framework Directive (Liska *et al*., 2008). During sampling from 15 August to 26 September 2007, 75 sites were sampled along the mainstream of the Danube River along its navigable way from river kilometre (rkm) 2600 near Ulm (DE) to the river mouth at rkm 0 (Kirschner *et al*., 2009) as shown in Fig. 1. In addition, 21 samples from the Danube's major tributaries and branches were included. At the most upstream sites, the Danube River is representative of a typical stream of the rhithron and characterized by its tributaries Iller, Lech and Isar (Kavka and Poetsch, 2002). The trip took 43 days which is a similar time period as the travel time of water in the Danube over this reach (see Velimirov *et al*., 2011). Samples were collected with sterile 1 L glass flasks from a water depth of approximately 30 cm. Glass flasks were sterilized by rinsing with 0.5% HNO_3_ and autoclaving them. For deoxyribonucleic acid (DNA) extraction of the particle‐associated bacterioplankton depending on the biomass concentration, 120–300 ml river water was filtered through 3.0 μm pore‐sized polycarbonate filters (Cyclopore, Whatman, Germany) by vacuum filtration. The filtrate, which represented the bacterioplankton fraction smaller than 3.0 μm (later referred to as ‘free‐living’ bacterioplankton), was collected in a sterile glass bottle and subsequently filtered through 0.2 μm pore‐sized polycarbonate filters (Cyclopore, Whatman, Germany). The filters were stored at −80°C until DNA extraction.

### DNA extraction and quantification of bacterial DNA using quantitative polymerase chain reaction

Genomic DNA was extracted using a slightly modified protocol of a previously published phenol‐chloroform, bead‐beating procedure (Griffiths *et al*., 2000) using isopropanol instead of polyethylene glycol for DNA precipitation. Total DNA concentration was assessed applying the Quant‐iT PicoGreen dsDNA Assay Kit (Life Technologies Corporation, USA), and 16S rRNA genes were quantified using Bacteria‐specific quantitative polymerase chain reaction (qPCR). Quantitative PCR reactions contained 2.5 μl of 1:4 and 1:16 diluted DNA extract as the template, 0.2 μM of primers 8F and 338 (Frank *et al*., 2007; Fierer *et al*., 2008) targeting the V1‐V2 region of most bacterial 16S rRNA genes and iQ SYBR Green Supermix (Bio‐Rad Laboratories, Hercules, USA). All primer information are available in Table S2. The ratios of measured 16S rRNA gene copy numbers in the different sample dilutions that deviated markedly from one after multiplication with the respective dilution factor were interpreted as an indicator for PCR inhibition.

### Preparation of 16S rRNA gene amplicon libraries

For the preparation of amplicon libraries, 16S rRNA genes were amplified and barcoded in a two‐step PCR procedure to reduce PCR bias that is introduced by long primers and sequencing adaptor overhangs (Berry *et al*., 2011). We followed the protocol as described by Sinclair and colleagues (2015). In short, 16S rRNA gene fragments of most bacteria were amplified by applying primers Bakt_341F and Bakt_805R (Herlemann *et al*., 2011; Table S2) targeting the V3‐V4 variable regions. In 25 μl reactions containing 0.5 μM primer Bakt_341F and Bakt_805R, 0.2 μM dNTPs (Invitrogen), 0.5 U Q5 HF DNA polymerase and the provided buffer (New England Biolabs, USA), genomic DNA was amplified in duplicate in 20 cycles. To use equal amounts of bacterial template DNA for increased comparability and reduction of PCR bias, the final volume of environmental DNA extract used for each sample was calculated based on 16S rRNA gene copy concentration in the respective sample determined earlier by qPCR (see above). For 105 samples, the self‐defined optimum volume of environmental DNA extract corresponding to 6.4 × 10^5^ 16S rRNA genes was spiked into the first step PCR reactions; however, for 27 samples, lower concentrations were used due to limited amounts of bacterial genomic DNA or PCR inhibition detected by quantitative PCR (see above). These 132 samples included eight biological replicates. Prior to the analysis, we removed four samples due to their extremely low genomic DNA concentrations and 16S rRNA gene copy numbers. Duplicates of PCR products were pooled, diluted to 1:100 and used as templates in the subsequent barcoding PCR. In this PCR, diluted 16S rRNA gene amplicons were amplified using 50 primer pairs with unique barcode pairs (Sinclair *et al*., 2015; Table S2). The barcoding PCRs for most samples were conducted in triplicates analogous to the first PCR (*n* = 100). The remaining 32 samples that had weak bands in first step PCR due to low genomic template DNA concentrations or high sample dilution were amplified in 6–9 replicates to increase amplicon DNA yield. Barcoded PCR amplicons were pooled in an equimolar fashion after purification using the Agencourt AMPure XP purification system (Beckman Coulter, Danvers, MA, USA) and quantification of amplicon concentration using the Quant‐iT PicoGreen dsDNA Assay Kit (Life Technologies Corporation, USA). Finally, a total of 137 samples including five negative controls resulted in four pools for sequencing.

### Illumina sequencing

The sequencing was performed on an Illumina MiSeq at the SciLifeLab SNP/SEQ sequencing facility hosted by Uppsala University. For each pool, the library preparation was performed separately following the TruSeq Sample Preparation Kit V2 protocol (EUC 15026489 Rev C, Illumina) with the exception of the initial fragmentation and size selection procedures. This involves the binding of the standard sequencing adapters in combination with separate Illumina‐specific multiplex identifier (MID) bar codes that enables the combination of different pools on the same sequencing run (Sinclair *et al*., 2015). After pooling, random PhiX DNA was added (5%) to provide calibration and help with the cluster generation on the MiSeq's flow cell.

### 16S rRNA gene amplicon data analysis

The sequence data were processed as outlined by Sinclair and colleagues (2015). After sequencing the libraries of 16S rRNA gene amplicons, the read pairs were de‐multiplexed and joined using the PANDAseq software v2.4 (Masella *et al*., 2012). Next, sequence reads (further referred to as ‘reads’) that did not bear the correct primer sequences at the start and end of their sequences were discarded. Reads were then filtered based on their PHRED scores. Chimera removal and OTU clustering at 3% sequence dissimilarity was performed by pooling all reads from all samples together and applying the uparse algorithm v7.0.1001 (Edgar, 2013). Here, any OTU containing less than two reads was discarded. Each OTU was subsequently taxonomically classified by operating a similarity search against the SILVAmod database and employing the CREST assignment algorithm (Lanzén *et al*., 2012). Plastid, mitochondrial and archaeal OTUs were removed. In addition, OTUs were also taxonomically annotated against the freshwater database (Newton *et al*., 2011) using the same method. If necessary, OTU rarefying for the purpose of standardizing sequence numbers was performed using the ‘rrarefy'‐function implemented in the r package vegan (Oksanen *et al*., 2013). For alpha diversity analysis (Chao1 richness estimator and Pielou's evenness), we rarefied down to 7000 reads per sample. This was based on one study revealing that for water samples, a sequencing depth of 5000 16S rRNA gene reads per sample captured more than 80% of the trends in Chao1 richness and Pielou's evenness (Lundin *et al*., 2012). Furthermore, this study could show that for water samples, 1000 reads per sample explained to 90% the trends in beta diversity (Bray–Curtis dissimilarity index). By rarefying down to 2347, which was the read number of the sample with the lowest reads, all samples could be included in the beta‐diversity analysis. Diversity measures, statistical analyses and plot generation were conducted in r (R Core Team, 2013) and using python scripts. The habitat index for the top 5000 OTUs was determined using the SEQenv pipeline (http://environments.hcmr.gr/seqenv.html). The SEQenv pipeline retrieves hits to highly similar sequences from public repositories (NCBI Genbank) and uses a text mining module to identify EnvO (Buttigieg *et al*., 2013) terms mentioned in the associated contextual information records (‘Isolation Source’ field entry for genomes in Genbank or associated PubMed abstracts). At the time of running SEQenv on our dataset (version 0.8), there were approximately 1200 EnvO terms organized into three main branches (namely, *environmental material*, *environmental feature* and *biome*). However, we used SEQenv to retrieve a subset of these terms, i.e. those that contain ‘Habitat’ (EnvO:00002036). Raw sequence data were submitted to the NCBI Sequence Read Archive under accession number SRP045083.

### General description of sequences

In total, DNA was extracted and sequenced from 132 filtered water samples originating from the Danube River and its tributaries. In addition, the same procedure was applied to five negative control samples. The sequencing yielded two 030 029 read pairs ranging from 3451 to 24 873 per sample. After quality filtering and mate pair joining as outlined in Sinclair and colleagues (2015), 1 572 361 sequence reads were obtained. The OTU clustering resulted in 8697 OTUs after the removal of all Plastid‐, Mitochondrion‐, *Thaumarchaeota*‐, *Crenarchaeota*‐ and *Euryarchaeota*‐assigned OTUs. Archaea‐assigned OTUs were removed because of the use of bacteria‐specific primers not giving a representative picture of the targeted Archaea community. The undesirable Plastid, Mitochondrion and Archaea sequences represented 19.1% of the reads and accounted for 625 OTUs. Next, for the alpha diversity analysis, we excluded any sample with less than 7000 reads, resulting in 8648 OTUs in the remaining 88 samples. By contrast, for the beta diversity analysis, which is less affected by rare OTUs, all samples were randomly rarefied to the lowest number of reads in any one sample in order to include a maximum number of samples in the analysis. This brought every sample down to 2347 reads, and any OTU containing less than two reads was discarded, which brought the total OTU count to 5082.

## Conflict of interest

The authors declare no conflict of interest.

## Supporting information


**Fig. S1.** Development of selected environmental parameters along the Danube River from the upstream region (rkm 2600; left) to the river mouth at the Black Sea (rkm 0; right). Left panel: alkalinity, pH, total bacterial production (TBP), total suspended solids (TSS); Right panel: nitrate (NO_3_^‐^), dissolved silicates (SiO_2_ diss) and phytoplankton biomass (Chl‐a) [PP biomass (Chl‐a)].
**Fig. S2.** Non‐metric multidimensional scaling plot of the compositional dissimilarities between communities (Bray–Curtis dissimilarities) of all samples of the Danube River and its tributaries. The stress value of the NMDS was 0.17. Dots represent free‐living bacterial communities (0.2–3.0 μm); triangles display particle‐associated bacterial communities (> 3.0 μm). Open symbols represent tributary samples, whereas full symbols indicate communities in the Danube River. The gradient from orange to blue via purple indicates the official Danube River kilometre assignment [rkm 2600 = upstream region near Ulm (DE), rkm 0 = river mouth at Black Sea] at the respective sampling site in the Danube River and for tributaries at the site (official rkm) of confluence with the Danube River, independent of its length.
**Fig. S3.** Box plot of variability in bacterial communities in different size fractions (0.2–3.0 μm and > 3.0 μm) based on beta‐dispersion of Bray–Curtis dissimilarities. Left: Variability (distance from centroid) in the free‐living bacterial community; Right: Variability in the attached bacterial community.
**Fig. S4.** Phylum‐level taxonomic composition of the bacterial communities along the Danube River. The Y‐axis shows the read proportions assigned to the five most abundant phyla in the free‐living fraction (left) and the particle‐associated fraction (right). Lower abundant phyla were included in the fraction ‘Others’ due to their low proportions. Samples are arranged from left to the right representing sequence from upstream (rkm 2600) to river mouth at the Black Sea (rkm 0).
**Fig. S5.** Results from the SEQenv analysis scoring sequences according to their environmental context (EnvO; environmental ontology). The Y‐axis represents the proportion of (A) ‘river’, (B) ‘lake’ and (C) ‘epilimnion’ terms associated with sequence reads per sample along the ‘mean dendritic stream length’ at each sampling site (X‐axis). Red dots indicate Danube River samples of the 0.2–3.0 μm size fraction (*n* = 42), and blue squares indicate samples from the > 3.0 μm size fraction (*n* = 52). Open dots and squares represent tributary samples of the free‐living and particle‐associated size fractions respectively. Dark blue lines represent fitted linear models for the Danube River samples with confidence intervals of 0.95 in red and blue for the respective fractions. Detailed regression statistics are given in Table S1.
**Fig. S6.** First occurrence plot of OTUs along the Danube River. Plotted are the numbers of OTUs occurring for the first time at the respective rkm of each sampling sites.
**Table S1.** Summary of regression statistics (slope, intercept, multiple R‐squared and *P*‐value) for fitted linear models between the proportion of different EnvO terms associated with sequence reads per sample and ‘mean dendritic stream length’ at the respective sampling site (upper section), and between alpha diversity measures [Chao1, Pielou's Evenness (J)] and geomorphological parameters (lower section). From the SEQenv analysis, regression statistics are given for the EnvO‐terms ‘groundwater’ (Fig. 6A), ‘soil’ (Fig. 6B), ‘river’ (Fig. S5A), ‘lake’ (Fig. S5B) and ‘epilimnion’ (Fig. S5C). FL: free‐living community of the Danube River (0.2–3.0 μm); **PA:** particle‐associated Danube River community (> 3.0 μm).
**Table S2.** List of used primers and barcodes for Illumina sequencing.
**Table S3.** Results of all measured environmental and chemical parameters during the JDS 2. Copy of the online available JDS2‐database‐content. Export date: 2013‐11‐07.
