# Supplementary material for: Bacterial diversity along a 2600 km river continuum
Source: Environ Microbiol. 2015 Jun 11;17(12):4994–5007. doi: 10.1111/1462-2920.12886 (PMC4918796; doi:10.1111/1462-2920.12886)
Supplement: Supplementary file 1 — Fig. S1. Development of selected environmental parameters along the Danube River from the upstream region (rkm 2600; left) to the river mouth at the Black Sea (rkm 0; right). Left panel: alkalinity, pH, total bacterial production (TBP), total suspended solids (TSS); Right panel: nitrate (NO3 ‐), dissolved silicates (SiO2 diss) and phytoplankton biomass (Chl‐a) [PP biomass (Chl‐a)]. Fig. S2. Non‐metric multidimensional scaling plot of the compositional dissimilarities between communities (Bray–Curtis dissimilarities) of all samples of the Danube River and its tributaries. The stress value of the NMDS was 0.17. Dots represent free‐living bacterial communities (0.2–3.0 μm); triangles display particle‐associated bacterial communities (> 3.0 μm). Open symbols represent tributary samples, whereas full symbols indicate communities in the Danube River. The gradient from orange to blue via purple indicates the official Danube River kilometre assignment [rkm 2600 = upstream region near Ulm (DE), rkm 0 = river mouth at Black Sea] at the respective sampling site in the Danube River and for tributaries at the site (official rkm) of confluence with the Danube River, independent of its length. Fig. S3. Box plot of variability in bacterial communities in different size fractions (0.2–3.0 μm and > 3.0 μm) based on beta‐dispersion of Bray–Curtis dissimilarities. Left: Variability (distance from centroid) in the free‐living bacterial community; Right: Variability in the attached bacterial community. Fig. S4. Phylum‐level taxonomic composition of the bacterial communities along the Danube River. The Y‐axis shows the read proportions assigned to the five most abundant phyla in the free‐living fraction (left) and the particle‐associated fraction (right). Lower abundant phyla were included in the fraction ‘Others’ due to their low proportions. Samples are arranged from left to the right representing sequence from upstream (rkm 2600) to river mouth at the Black Sea (rkm 0). Fig. S5. Result [file EMI-17-4994-s001.zip › Figure.S5_SuppInfo.pdf]

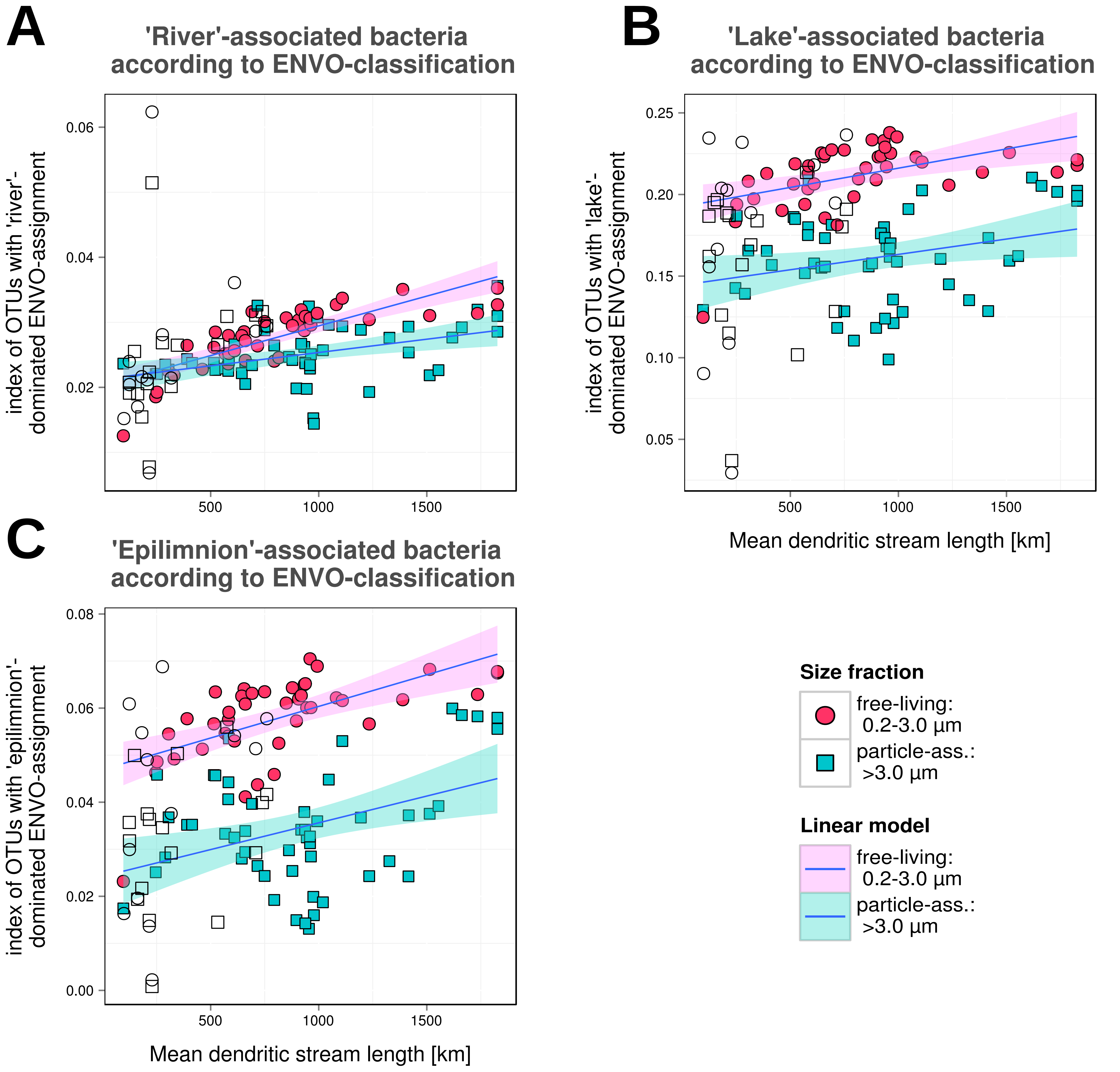
A
'River'-associated bacteria according to ENVO-classification
index of OTUs with 'river'-dominated ENVO-assignment
0.06
0.04
0.02
500
1000
1500
B
'Lake'-associated bacteria according to ENVO-classification
index of OTUs with 'lake'-dominated ENVO-assignment
0.25
0.20
0.15
0.10
0.05
500
1000
1500
Mean dendritic stream length [km]
C
'Epilimnion'-associated bacteria according to ENVO-classification
index of OTUs with 'epilimnion'-dominated ENVO-assignment
0.08
0.06
0.04
0.02
0.00
500
1000
1500
Mean dendritic stream length [km]
Size fraction
free-living: 0.2-3.0 µm
particle-ass.: >3.0 µm
Linear model
free-living: 0.2-3.0 µm
particle-ass.: >3.0 µm
